# Barriers to HPV immunization among blacks and latinos: a qualitative analysis of caregivers, adolescents, and providers

**DOI:** 10.1186/s12889-016-3529-4

**Published:** 2016-08-25

**Authors:** Ingrid T. Katz, Laura M. Bogart, Chong Min Fu, Yingna Liu, Joanne E. Cox, Ronald C. Samuels, Tami Chase, Pamela Schubert, Mark A. Schuster

**Affiliations:** 1 Department of Medicine, Brigham and Women’s Hospital, Boston, Massachusetts USA; 2 Massachusetts General Hospital, Center for Global Health, Boston, MassachusettsMA USA; 3 Harvard Medical School, Boston, Massachusetts USA; 4 Division of General Pediatrics, Boston Children’s Hospital, Boston, Massachusetts USA; 5 RAND Corporation, Santa Monica, California USA; 6 Division of Women’s Health, 1620 Tremont Street, 3rd Floor BWH, Boston, MA 02120 USA

**Keywords:** HPV immunization, Adolescents, Parental preferences, Provider preferences, Qualitative methods

## Abstract

**Background:**

Despite recommendations that 11–12-year-olds receive the full three-shot Human papillomavirus (HPV) vaccine series, national HPV immunization coverage rates remain low. Disparities exist, with Blacks and Latinos being less likely than Whites to complete the series. We aimed to identify and compare barriers to HPV immunization perceived by healthcare providers, Black and Latino adolescents, and their caregivers to inform a clinic-based intervention to improve immunization rates.

**Methods:**

We conducted semi-structured interviews between March and July 2014 with Black and Latino adolescents (*n* = 24), their caregivers (*n* = 24), and nurses (*n* = 18), and 2 focus groups with 18 physicians recruited from two pediatric primary care clinics. Qualitative protocol topics included: general perceptions and attitudes towards vaccines; HPV knowledge; and perceived individual and systems-level barriers affecting vaccine initiation and completion.

**Results:**

Themes were identified and organized by individual and systems-level barriers to HPV immunization. Adolescents and their caregivers, particularly Blacks, expressed concerns about HPV being an untested, “newer” vaccine. All families felt they needed more information on HPV and found it difficult to return for multiple visits to complete the vaccine series. Providers focused on challenges related to administering multiple vaccines simultaneously, and perceptions of parental reluctance to discuss sexually transmitted infections.

**Conclusions:**

Optimizing HPV immunization rates may benefit from a multi-pronged approach to holistically address provider, structural, and individual barriers to care. Further research should examine strategies for providing multiple modalities of support for providers, including a routinized system of vaccine promotion and delivery, and for addressing families’ concerns about vaccine safety and efficacy.

## Background

Human papillomavirus (HPV) infection is the most common sexually transmitted infection (STI) in the United States (U.S.), and each year, an estimated 26,000 new cancers attributable to oncogenic HPV are diagnosed in women and men [1]. Recent U.S. population-based studies conducted by the Centers for Disease Control and Prevention (CDC) show that 66 % of cervical cancers, 55 % of vaginal cancers, 79 % of anal cancers, and 62 % of oropharyngeal cancers are attributable to oncogenic HPV types 16 or 18 [2]. Disparities exist, with higher rates of HPV-associated cancers among American Indian/Alaskan Native, Black, and Latina women than among Whites [3].

National HPV immunization coverage rates remain remarkably low, despite recommendations from the Advisory Committee on Immunization Practices and the American Academy of Pediatrics to routinely provide immunization of girls and boys aged 11 or 12 years [4–6], starting in 2006 for girls and 2011 for boys. Available HPV vaccines are provided in a 3-dose series [7–12]. In mid-2014, only 57.3 % of girls aged 13–17 in the U.S. had initiated the three-dose series and only 37.6 % had completed it, and only 20.8–34.6 % of boys had received ≥1 HPV dose [13]. These figures are particularly striking when compared with immunization rates for other adolescent vaccines (e.g., tetanus, diphtheria, and acellular pertussis vaccine [Tdap] and meningococcal conjugate vaccine [MenACWY]), which range from 74.0 % for MenACWY up to 86.0 % for Tdap [13]. Moreover, Black female adolescents who initiate the vaccine have significantly lower rates of HPV 3-dose series completion than do White and Latina female adolescents, and overall rates of immunization remain lower among boys than girls [13].

Adolescent vaccines present some distinct challenges compared to childhood vaccines, including the fact that adolescents may be involved in decision-making, in addition to caregivers and providers [14–16]. There are also specific challenges for HPV immunization, including its association with adolescent sexual activity and STI acquisition [17], the lack of a school requirement [18], and misperceptions about vaccine efficacy and safety [19].

Recent research has shown that provider recommendations and communication with caregivers are an essential step in HPV vaccine uptake and completion [20, 21]. Yet, few studies to date have focused on understanding how perceived barriers to HPV vaccination by race may reduce uptake. Data are critical to providing information to optimize effective multi-tiered interventions, in order to achieve the goal of *Healthy People 2020,* which is to reach an HPV vaccination rate of 80 % of adolescents aged 13–15 years by 2020 [13, 22]. Therefore, we conducted a qualitative study of Black and Latino caregiver-adolescent dyads, as well as their pediatric healthcare providers, to assess their perspective on barriers to HPV immunization and the impact of these barriers on uptake of the vaccine. Our goal was to use the findings to inform development of an evidence-based intervention to improve HPV immunization rates among adolescent boys and girls. In addition, we performed a concordance analysis to compare the extent to which caregiver-adolescent pairs agreed about their perceptions of the HPV vaccine.

## Methods

### Study site

We performed a qualitative study between March and July 2014, using focus groups with physicians and nurse practitioners and semi-structured interviews with nurses, parents and other caregivers (will be referred to as caregivers henceforth), and adolescents who accessed care at either of two clinics. One is housed on the campus of a children’s hospital and cares for over 14,000 young people, drawing mostly from Boston’s low-income neighborhoods (65 % are Medicaid beneficiaries). It serves a racially and ethnically diverse population of patients, who are 45 % Black, 45 % Latino, and 10 % White. In addition, 15 % are non-English-speaking. The other is a community health center that provides cares for over 4,000 pediatric patients, drawn primarily from the surrounding low-income communities and other similar communities where prior local residents have moved. Eighty percent are Medicaid beneficiaries. The patients are 70 % Latino, 20 % Black, and 10 % White, with many recent immigrants (e.g., Dominican) and refugees (e.g., Somali). It primarily serves a Spanish-speaking population.

Both clinics use the same advanced electronic health records. As of September 2013 (the date when funding was received for this study), 62 % of patients had received at least one dose of the HPV vaccine by their 13^th^ birthday (females 73 % and males 49 %), and 23 % had completed the three-dose series (females 35 % and males 9 %).

### Sampling and recruitment

We performed 24 semi-structured interviews with adolescent-parent pairs (totaling 48 individual interviews). We used a purposive sampling strategy to ensure adequate representation of Black and Latino families [23]; specifically, we selected a sample of adolescents split between Black and Latino background, the two dominant groups in the clinics. Within each group, we chose a random sample of potential participants, stratified by gender and the number of vaccine doses completed (0, 1, or 2), to ensure we engaged with a diverse array of participants. Forty-three percent of those approached agreed to participate in this study. We also interviewed 18 nurses and conducted 2 focus groups with 18 physicians and a nurse practitioner in order to gain an understanding of how the HPV vaccine was being offered to adolescent patients and their caregivers and of what strategies might be effective to increase vaccination rates.

### Eligibility criteria

Adolescents were eligible if they were 12–17 years-old, were a patient at either of the two study sites, and had completed zero, one, or two doses of the HPV vaccine series. Caregivers were eligible if their adolescent child was participating and they accompanied the adolescent. Adolescents provided written assent and caregivers provided written informed consent. All healthcare providers who worked at the sites were eligible to participate; they provided oral consent.

### Data collection and preparation

Data were collected via semi-structured, 30–60 min in-depth interviews with adolescents and caregivers, and two 60-min focus groups with healthcare providers. Caregivers and adolescents were each provided $50 for their participation. Both the semi-structured interviews and focus groups were designed to elicit information about perceived parent and patient barriers to HPV immunization and potential intervention components to address these barriers. The parent and adolescent protocols additionally included questions to assess knowledge about HPV and the HPV vaccine, while providers were questioned about their perceptions of patients’ and parental knowledge.

Qualitative protocol topics included: (1) General perceptions and attitudes towards vaccines; (2) HPV knowledge; (3) Perceptions of the HPV vaccine; (4) Perceived systems-level barriers and facilitators affecting vaccine uptake and adherence; and (5) Perceived individual-level barriers and facilitators affecting vaccine uptake and adherence (see Table 1). A trained research assistant conducted interviews in English or Spanish, based on participant preference (half of the interviews with the Latino caregivers were conducted in Spanish). All interviews were audio-recorded (with permission), and were transcribed and translated into English as necessary.

**Table 1:** Qualitative protocol for understanding attitudes and experiences with the HPV vaccine

Patients and Parents (Semi-Structured Interviews)		Providers (Focus Groups)
Question topic	Question example	Question Example
*Patients and Parents* General perceptions and attitudes towards vaccines *Providers* Perceptions of patient/parent perceptions	• How do you feel about vaccines in general? • To what extent do you think that vaccines are helpful (or effective)? Do you think that some vaccines are more helpful or effective than others? If so, which ones? • To what extent do you think that vaccines are harmful (or ineffective)? Do you think that some vaccines are more harmful than others? If so, which ones? • Whom do you trust to give you information about vaccines in general? • Whom do you usually go to first when you have questions about vaccines, and why? (Probe for people you know, from healthcare providers, from others, and from the internet or media)	• How much do you think parents and adolescent patients trust or do not trust the HPV vaccine to protect adolescents’ health? • What types of concerns have parents [adolescents] said to you about the HPV vaccine? What types of positive feedback have you heard?
*Patients and Parents* Assessment of HPV knowledge *Providers* Assessment of patients’ and parental knowledge	• Tell me what comes to mind when you hear “HPV?” • How is HPV prevented, if at all? • How did you learn about HPV? • Which sources were most helpful? Least helpful?	• To what extent do you think they think that the HPV vaccine is harmful, or ineffective? To what extent do they think it is safe or effective? • To what extent do you think they think that the HPV vaccine is necessary? • To what extent do you think they think that the HPV vaccine does or does not have side effects? • To what extent do you think they think that the HPV vaccine does or does not cause discomfort when or after it is administered? • To what extent do you think they think that the vaccine does or does not cause teens to have sex? • To what extent do you think they think that the HPV vaccine is helpful (or effective)? • To what extent do you think that they know that the HPV vaccine prevents cancer? Prevents STIs?
*Patients and Parents* Perceptions of the HPV vaccine *Providers* Perceptions of how patients and parents view the HPV vaccine	• How much do you trust or not trust the HPV vaccine? Why or why not? • To what extent are you willing or not willing to vaccinate your child(ren) for HPV? • To what extent do you think that the HPV vaccine is necessary or not necessary for boys? For girls? • To what extent do you think that the HPV vaccine is harmful or ineffective? • From whom or where have you heard information about the HPV vaccine? • To what extend do you believe what you have heard about the HPV vaccine? • From whom or where do you think your child gets information about HPV?	• How do patients and parents react to the idea of the HPV vaccine? • What kinds of things do parents typically say when you bring up the vaccine? • What kinds of questions do parents ask? • How are these questions addressed? By whom? • What types of reasons do patients and parents give when declining the vaccine? • How do you respond when parents decline for their adolescent to get the vaccine? • In general, how do you address a situation where a parent declines? What do you say?
*Patients and Providers* Perceived systems-level factors affecting vaccine uptake and adherence *Providers* Perceptions of provision of care and systems-level factors influencing vaccine uptake and adherence	• If you have not received the vaccine, what factors played a role? • What factors related to you, your child, or your healthcare provider played a role? • How much do you intend to get the 2^nd^ and 3^rd^ shots for your child? • What kinds of things might be a barrier to your child coming back to get the 2nd and 3rd shots? • What kinds of things might be helpful in getting your child back for the 2nd and 3rd shots?	• What kinds of things do you think that the clinic does particularly well and what kinds of things do you think that the clinic does less well in getting adolescents to take the HPV vaccine? • Why do you think that things work that way in the clinic? • What kinds of things might be a barrier to adolescent patients coming back to get the 2^nd^ and 3^rd^ shot? • What kinds of things might be helpful in getting adolescent patients back for the 2^nd^ and 3^rd^ shots? • What kinds of things can healthcare providers and clinics do to help get more children vaccinated for HPV? • What kinds of information could healthcare providers give to parents about the vaccine? • How should healthcare providers communicate with parents to let them know about the vaccine? • What would help parents and adolescents initiate the vaccine series? • What would help parents and adolescents complete the vaccine series? • What kinds of things can the clinic do to help remind parents about the 2^nd^ and 3^rd^ doses of the vaccine? • What methods of communication reminders would be most helpful (email, texts, phone, etc.)?
*Patients and Parents* Perceived individual-level factors affecting vaccine uptake and adherence *Providers* Perceptions offering the HPV vaccine	• What kinds of questions and concerns did you have about the HPV vaccine when it was first introduced to you by the clinic staff? • What kinds of questions and concerns did your child have? • To what extent were your and your child’s concerns addressed or not addressed? If yes, how and by whom, and with what information? If no, why do you feel that your concerns were not addressed? • If you did receive the vaccine, how was it decided that you would get the HPV vaccine?	• How do you discuss HPV with adolescent patients and their parents? • What factors influence your decision to offer the HPV vaccine to an adolescent and parent of an adolescent patient? • How do you present information about the HPV vaccine to patients and parents? • What kind of things do you typically discuss with adolescents about the HPV vaccine? • When offering the vaccine, to what extent do you talk to the adolescent?
*Patients and Parents* Feedback on proposed intervention *Providers* Feedback on proposed intervention	• What kinds of things can healthcare providers and clinics do to help get more children vaccinated for HPV? • What do you think about the following intervention components: • Text message reminders for follow-up visits? • Educational materials like pamphlets and videos? • Computer systems to track patients who do and do not initiate and complete the vaccine? • Tracking whether physicians are offering and giving the vaccine, and giving them feedback about how they are doing? • Provider reminders about patients who are due to start the vaccine series or receive another dose? • Teaching healthcare providers how to communicate with patients who refuse the vaccine?	• What kinds of things can healthcare providers and clinics do to help get more children vaccinated for HPV? • What do you think about the following intervention components: • Text message reminders for follow-up visits? • Educational materials for parents and adolescents like pamphlets? • Computer systems to track adolescent patients who do and do not initiate and complete the vaccine? • Tracking whether physicians are offering and giving the vaccine, and giving them feedback about how they are doing? • Implementing and using a quality measure of clinic and physician HPV vaccination rates? • Provider reminders in the electronic health record about adolescent patients who are due to start the vaccine series or receive another dose? • Teaching healthcare providers how to use supportive communication strategies to address vaccine refusal?

### Conceptual framework

Members of our team developed a conceptual model to guide socio-behavioral research on HPV vaccination uptake and completion [24]. This theoretical framework, titled ‘The Vaccine Perceptions, Acceptability and Adherence Model,’ is based on a systematic review of the literature, and provided the basic theoretical framework for our qualitative protocol. The constructs in this model – including perceived risk, perceived effectiveness of the vaccine, perceived barriers to vaccine uptake and completion, and cues to action (situational factors that trigger one to get vaccinated or adhere to a vaccine regimen) – have been previously shown to be important predictors of vaccination [24]. The model provides an integrated and dynamic framework that recognizes cultural and economic forces at play in settings of economic adversity. The model recognizes the dynamic exchanges among patients, their caregivers, and their providers, that are necessary for vaccine-related decision-making. Based on this model, we developed our qualitative interview guide, using open-ended questions that allowed for inductive analyses.

### Data analysis

Four team members (ITK, LMB, CMF, and YL) served as coders and began an inductive analysis with a detailed review of all transcripts. We used a 3-stage analytic strategy with open and axial coding, followed by selective coding, to reflect relationships among codes [25]. We developed a codebook, organized according to the hierarchy developed through axial coding, as part of our deductive phase of coding. For this phase, we were guided by our research question. We used Dedoose Version 5.0.11 web application for managing, analyzing, and presenting our qualitative research data (2014, Los Angeles, CA: SocioCultural Research Consultants, LLC, www.dedoose.com). We assigned labels to each category and identified illustrative quotes from interview transcripts. We ensured trustworthiness of the data by having two authors independently code the same 20 % of transcripts (CMF, YL). Inter-rater consistency was very good on all major themes (ranging between 71.8 % and 84.6 %) [26]. We then examined the distribution of themes within and among participant groups, by comparing and contrasting stakeholder groups – specifically providers, caregivers, and adolescents.

A primary goal of the study was to understand relationships between caregivers’ and adolescents’ views on the HPV vaccine. To do this, we performed a concordance analysis [27, 28] to reflect the match between parental and adolescent perceptions. Concordance analyses were conducted across themes that had a finite number of response choices and that were asked of nearly all caregivers and adolescents. We then identified illustrative quotes to support these findings.

## Results

Eighty-four individuals participated (24 caregivers, 24 adolescents, 18 doctors, and 18 nurses). Adolescent and caregiver participants are described in Table 2. Ninety-two percent of the caregivers were parents (22/24) and 2 were grandmothers. Themes were identified and organized by individual-level barriers and systems-level barriers to HPV immunization. Within each barrier type, we compared perceptions among providers, adolescents, and their caregivers to assess for differences among stakeholders and across racial/ethnic groups. Themes identified in our analyses are detailed below, with quotes in Tables 3 and 4. Experiences were generally consistent across the two clinical settings.

**Table 2:** Adolescent and Caregiver Participants (*n* = 48)

Characteristic	Black^a^	Latino^a^	*P*-value
Caregivers			
Age			0.803
Mean (SD)	40.44 (13.62)	39.14 (8.61)	
Range	31-75	30-59	
Education			0.067
Grades 1-6	0	2	
Grades 7-11	1	1	
High school graduate	1	6	
Some college, no degree	6	1	
College degree	2	2	
Some graduate	0	1	
Graduate degree	1	1	
Marital status			0.869
Single	5	5	
Married	4	7	
Partner	2	2	
Adolescents			1
Gender			
Female	6	7	
Male	6	7	
Age			0.331
Mean (SD)	13.67 (1.61)	13.07 (1.44)	
Range	12-16	12-16	
Series status			0.549
0 doses	7	6	
1 dose	3	7	
2 doses	1	1	
3 doses	1	0	

^a^One parent and two adolescents identified as Black and Latino. Numbers are reflected accordingly in the table

**Table 3:** Individual and Structural-level Barriers to HPV Immunization

Category	Row #	Participant	Representative Quotation
Mistrust			
Caregiver perceptions	1	Black caregiver of an adolescent girl	“Well, the earlier vaccines – the measles and mumps and all that – I am fine with those… It’s the newer ones that I am not.”
Caregiver perceptions	2	Black caregiver of an adolescent girl	“We don’t know what’s inside of the vaccines, and they can be harmful if you don’t know what it is. It might not mix well with bodies and cells. They should just be more careful. It is like we’re being picked like guinea pigs.”
Adolescent perceptions	3	Latino adolescent boy	Interviewer: “Do you trust the vaccine?” Adolescent: “Not at all…because I don’t know what it does…I never heard it…it depends how big the needle is.”
Lack of education			
Adolescent perceptions	4	Latina adolescent girl	“They usually just give you a handout, like a little sheet explaining what it is and the side effects and what could possibly happen. And so, that is it. I really don’t know much about it.”
Caregiver perceptions	5	Black caregiver of an adolescent girl	“I didn’t know that I needed to come back. I had no clue that you had to give me more than one, and I wasn’t told that at the time.”
Healthcare provider perceptions	6	Physician	“With HPV—a lot of them haven’t heard about it… A lot of times I look at the adolescents and say have you seen those ads on TV—Gardasil? They will be the ones who recognize it. Their parents, not so much.”
Challenges associated with the dosage schedule			
Healthcare provider perceptions	7	Nurse	“I think getting them back for their second and third is a real problem. It would be nice if we had a better system to remind parents… I find that a lot of times you see them and they get their second [shot] a year later. They come then because the adolescents only come once a year.”
Lack of Routinization			
Healthcare provider perceptions	8	Physician	“We’re under the gun in terms of time. So if our primary directive at a visit is to get these kids up-to-date with immunizations, then the goal is to sort of get that done with as little conversation and resistance as possible, and then hopefully you have time to have meaningful conversations about real-world stuff like with what’s going on with sexual activity or dating or boyfriends or parent relationships or whatever else the myriad of things that are going on. So all these conversations that we are having around HPV are really—we’re just trying to sell it, for lack of a better word. We’re just trying to get it done so that we can move on to the more meaningful points of the visit.”
Administration of multiple vaccines at the same visit			
Healthcare provider perceptions	9	Nurse	“I think that, you know, that offering it to children at eleven and just sort of matter of fact tell them this is what we do at eleven, along with their Tdap and their meningococcal vaccine. It works pretty well.”

**Table 4:** Representative Quotes from Concordance Analysis of Caregiver-Adolescent Dyads

Category	Row #	Adolescent	Caregiver
Lack of education (concordance)	1	I: “What factors played a role in your decision not go get the HPV vaccine.” R: “Umm… me not knowing about it. Not knowing enough.” I: “And that’s the only thing was the lack of information?” R: “Yeah.”	“Just because like I said, [the physician] could have given me more information. Then I could’ve read up on it even if I wasn’t going to let him get it anyway. So, it wouldn’t have mattered, but still, I would’ve still wanted to know what it is and I think that that’s the problem that they create these new vaccines and they don’t let the parents know about them, read about them before the scheduled appointment and so when the appointment comes it’s like no, and this is why I’m saying no.”
Mistrust (concordance)	2	“Yeah, [my caregiver] talked to the doctor, that’s why she said, ‘Oh I don’t know if I want [me] to get it.’ Because there’s a thing that it said it killed people—whatever.”	“To be honest, death wasn’t something I thought about a lot. It wasn’t until I just read that article--how true it is, I don’t know. Supposedly that was the stats that they had, and there were a lot of deaths in it, among other things. But I—I think my mind won—because being paralyzed was one of the other things. [My child] could become paralyzed, it could be temporary and could be permanent… It was that part of that, you know what I mean?”
Association with pain (discordance)	4	“No, I was just scared of getting it because I haven’t had a shot in a while, so…”	“Well, my daughter’s first question was, ‘Is it going to hurt,’ but, other than that, no [problem].”

### Individual-level factors

#### Overview

Two individual-level barriers to care emerged in the data: mistrust of vaccines, and lack of education about vaccine efficacy and safety. Vaccine mistrust was a common theme among Black adolescents and their caregivers, who expressed concerns about HPV being a “newer” vaccine and potentially untested. Families also felt they needed more education from providers on the importance of HPV immunization; however, providers were often reluctant to discuss the vaccine in detail due to perceived concerns about the sexual association of the virus.

1. Mistrust:
  ***Adolescent and caregiver perceptions.*** Black caregivers discussed their mistrust of vaccine safety in general, with less focus on the HPV vaccine in particular. Caregivers who expressed mistrust of vaccines tended to speak about “newer” vaccines being less trustworthy than vaccines that they received in their childhood (Table 3, Row 1), and the risk of “experimentation” on their children (Table 3**,** Row 2). While adolescents had less mistrust of vaccines, they acknowledged a generalized fear of vaccines related to potential pain from an injection. This view was not specific to the HPV vaccine (Table 3, Row 3). In particular, individuals who had not yet received the vaccine tended to discuss the role of mistrust in leading them to avoid vaccination, as compared to those who had initiated the series.
2. Lack of education:
  ***Adolescent and caregiver perceptions***. Adolescents and caregivers believed that they lacked adequate information to make informed decisions about the vaccine. In particular, parents of boys spoke frequently about their mistaken belief that boys needed vaccination only to protect potential future female partners. Caregivers reported that nurses and physicians did not provide adequate explanations of the vaccine’s safety and efficacy to allow families to make informed decisions. Caregivers and adolescents were often uncertain about whether they needed to come back to complete the series (Table 3, Rows 4 and 5).
  ***Healthcare provider perceptions*.** Healthcare providers discussed concerns that they had about discussing the HPV vaccine, given its association with an STI (Table 3, Row 6). Although all physicians recognized the importance of the HPV vaccine, many perceived caregiver resistance to the vaccine and thought it was likely due to concerns about promoting sexual intercourse. Providers sometimes acknowledged that they deferred potentially challenging conversations about the vaccine to subsequent visits.

### Systems-level factors

#### Overview

We identified three systems-level barriers to care in the qualitative data: challenges associated with the dosage schedule, lack of standardization of delivery and reminder process, and administration of multiple vaccines at the same visit.

1. Challenges associated with the dosage schedule:
  ***Healthcare provider perceptions.*** Both physicians and nurses discussed the challenges in requiring adolescents to return for the second vaccination at 2 months and the third vaccination at 6 months after the first dose because the timing did not match other standard clinical visits (Table 3, Row 7). Providers felt this resulted in large numbers of adolescents who had not completed all 3 doses of the vaccine.
2. Lack of routinization:
  ***Healthcare provider perceptions.*** Providers described feeling burdened with checklists and reminders about many elements of healthcare maintenance, and the challenge to monitor HPV series completion. This concern was often discussed in association with the need for an increased number of support staff to facilitate communication and collaboration, and a lack of system-level reminders. Physicians found themselves relying on nurses to help remind them to offer the HPV vaccine, or to initiate a dialogue with patients and caregivers. Many physicians and nurses described having inadequate time in a clinic visit to address all the needs of their patients (Table 3, Row 8).
3. Administration of Multiple Vaccines at the same visit:
  ***Healthcare provider perceptions****:* Many providers described concerns about the number of vaccines being administered to eleven-year-olds. Physicians noted that there are already two standard vaccines given at that visit (Tdap and MenACWY) and a “third shot will put the kids at that age over the edge,” on top of yet another vaccine (the influenza vaccine) during several months of the year. Some nurses, though, felt that a third vaccine could be incorporated into an annual visit in a manner that would not pose additional challenges (Table 3, Row 9). Concerns about receiving multiple vaccines simultaneously did not factor prominently in the interviews of caregivers and adolescents.

### Caregiver-adolescent concordance analysis

We identified three themes through our concordance analysis: Lack of education, mistrust, and association with pain. Caregivers and adolescents were consistently concordant within dyad in discussing how they lacked information and adequate education on the HPV vaccine, which often translated into a reluctance to accept the vaccine when offered (Table 4, Row 1). In addition, caregivers and adolescents in the same dyad consistently stated that they mistrusted the vaccine, which was cited as a reason both for initial refusal of the vaccine and concerns about returning to complete the 3-shot series (Table 4, Row 2). Conversely, adolescents were much more concerned about the potential for pain associated with the vaccine than were their caregivers. There was little discussion among either caregivers or adolescents of the structural barriers cited by providers, including dosage schedule, lack of routinization, and the number of vaccines administered at a given visit.

## Discussion

Interviews and focus groups with providers and families revealed a broad range of challenges that they face, respectively, in administering and completing the HPV vaccine series. Providers primarily focused on systems-level challenges related to standardization of vaccine administration, time pressures in the clinic, and the need to optimize collaboration among providers. They also discussed their concerns related to potentially negative perceptions of the vaccine from caregivers. This led to avoidance of what they anticipated to be challenging discussions (e.g., discussing the transmission of HPV through sexual contact) in order to cover most age-appropriate issues within what they described as tight time constraints available for visits.

Caregivers and adolescents, conversely, rarely mentioned systems-level barriers to care. In general, most caregivers and adolescents focused primarily on individual-level barriers, with both groups expressing concern over the lack of education they received regarding vaccine safety and efficacy. In addition, adolescents discussed fears of potential pain associated with the vaccine, and caregivers describing mistrust of the vaccine. Black caregivers, in particular, expressed concern over HPV being a “newer” vaccine, and therefore potentially experimental in nature. Mistrust also appeared to be a barrier to vaccination among those who had never received the vaccine. Certain constructs identified through our qualitative research map onto our conceptual model [24]. In particular, patients and their families expressed the need to weigh the perceived risk of receiving a vaccine against the perceived effectiveness of the vaccine. Participants who expressed significant mistrust tended to express more concern about the perceived risk of the HPV vaccine.

Prior literature has suggested an association between medical mistrust and disparities in a variety of health outcomes [29–33]. While fewer studies have focused on understanding the link between medical mistrust and prevention of STIs, recent research has shown that women with higher mistrust were less likely to have engaged in preventive health behaviors such as HPV immunization, a trend that was exacerbated when patients and providers were racially discordant [34]. This finding has significant public health implications given higher rates of HPV prevalence and cervical cancer incidence and mortality and lower rates of HPV vaccine completion in Black and Latina women, compared to White women [35–37].

The concordance analyses revealed that both caregivers and adolescents in the same family were consistent in feeling that they lacked adequate education and information regarding the vaccine to make sound decisions. Many expressed that this was the primary reason for not obtaining a shot at a given visit. Caregivers and adolescents also described how vaccine mistrust factored into their decision (particularly among caregivers), and concerns about potential pain associated with the shot (particularly among adolescents). Overall, families rarely mentioned structural barriers to vaccination, whereas providers listed these factors as primary reasons to avoid administering the vaccine. This is supported by recent literature showing that primary care providers often perceive discussions about the HPV vaccine to be time-consuming and necessitating more parental engagement than discussions about other mandatory vaccines [38].

Given these findings, several potential strategies may be useful for improving rates of HPV vaccine immunization. Research supports a varied and flexible approach to intervention design, which can be implemented within healthcare settings to increase HPV vaccine uptake across diverse populations [39]. For example, standardization of work-flow using electronic health record reminders could decrease the burden on individual providers. In addition, longer vaccination appointments could be scheduled to educate families about what vaccines children should have, while providing adequate information focused on vaccine safety, efficacy, and misinformation. Educational messaging focused on addressing mistrust could potentially incorporate research findings suggesting the HPV vaccine is not linked to risky sexual behaviors [40].

This study is subject to a key limitation - all interviews were conducted with participants from one healthcare organization, albeit two distinct sites within the system. Thus, our findings may not be representative of practices nationwide. The strength of qualitative research, however, lies in its ability to explore a range of viewpoints on a given topic, as opposed to ensuring generalizability. To optimize our potential to engage with a range of individuals, we recruited our population from a community-based health center and an academic medical center and included boys and girls, as well as their caregivers and providers.

## Conclusions

Our findings provide an in-depth examination of the many barriers to HPV immunization among traditionally underserved communities and highlight the need to design interventions that effectively address both structural and individual barriers to care. Further research could examine the impact of incorporating multiple modalities of support for providers, including a routinized system of vaccine promotion and delivery while addressing families' concerns about vaccine safety and efficacy.
